# Blood oxygen saturation is lower in persons with pre-diabetes and screen-detected diabetes compared with non-diabetic individuals: A population-based study of the Lolland-Falster Health Study cohort

**DOI:** 10.3389/fepid.2022.1022342

**Published:** 2022-10-13

**Authors:** Jens Christian Laursen, Randi Jepsen, Neda Esmailzadeh Bruun-Rasmussen, Marie Frimodt-Møller, Marit Eika Jørgensen, Peter Rossing, Christian Stevns Hansen

**Affiliations:** ^1^Complications Research, Steno Diabetes Center Copenhagen, Herlev, Denmark; ^2^Center for Epidemiological Research, Nykøbing Falster Hospital, Nykøbing Falster, Denmark; ^3^Steno Diabetes Center Greenland, Nuuk, Greenland; ^4^Department of Clinical Medicine, University of Copenhagen, Copenhagen, Denmark

**Keywords:** hypoxia, microvascular complications, albuminuria, type 2 diabetes, pre-diabetes

## Abstract

**Aims:**

Low blood oxygen saturation is associated with increased mortality and persons with diabetes have sub-clinical hypoxemia. We aimed to confirm the presence of sub-clinical hypoxemia in pre-diabetes, screen-detected diabetes and known diabetes.

**Methods:**

Pre-diabetes was defined as hemoglobin A1C (HbA_1C_) ≥ 42 mmol/mol and <48 mmol/mol; known diabetes as history or treatment of diabetes; screen-detected diabetes as no history or treatment of diabetes and HbA_1C_ ≥ 48 mmol/mol. Blood oxygen saturation was measured with pulse oximetry. Urine albumin-to creatinine ratio (UACR) was measured on a single spot urine.

**Results:**

The study included 829 adults (≥18 years) with diabetes (713 (86%) with known diabetes; 116 (14%) with screen-detected diabetes) and 12,747 without diabetes (11,981 (94%) healthy controls; 766 (6%) with pre-diabetes). Mean (95% CI) blood oxygen saturation was 96.3% (96.3% to 96.4%) in diabetes which was lower than in non-diabetes [97.3% (97.2–97.3%)] after adjustment for age, gender, and smoking (*p* < 0.001), but significance was lost after adjustment for BMI (*p* = 0.25). Sub-groups with pre-diabetes and screen-detected diabetes had lower blood oxygen saturations than healthy controls (*p*-values < 0.01). Lower blood oxygen saturation was associated with higher UACR.

**Conclusions:**

Persons with pre-diabetes and screen-detected diabetes have sub-clinical hypoxemia, which is associated with albuminuria.

## Introduction

Low blood oxygen saturation is associated with increased mortality (1), and persons with diabetes have low blood oxygen saturation compared with non-diabetic controls. This has been shown in type 1 diabetes (2, 3), but findings from a small study in type 2 diabetes also indicated lower blood oxygen saturation compared with non-diabetes, although the difference was not statistically significant (4), The demonstrated differences compared with non-diabetic controls were small (type 1 diabetes vs. non-diabetic controls: −0.5%; type 2 diabetes vs. non-diabetic controls: −0.3%), but due to the oxygen-hemoglobin dissociation curve, small differences in peripheral blood oxygen saturation could imply large differences in peripheral tissue oxygen partial pressure (5). The exact mechanisms behind lower blood oxygen saturation in diabetes are unknown, but reduced pulmonary diffusion capacity as a consequence of pulmonary microvascular damage could play a role (3). Other potential causes are impaired lung function (6), pulmonary co-morbidities such as chronic obstructive lung disease (COPD) or asthma, or impaired red blood cell function. Previous studies demonstrating sub-clinical hypoxemia in persons with diabetes were small and did not include persons with pre-diabetes or screen-detected diabetes. Population-based studies are needed to confirm previous findings and investigate potential differences in blood oxygen saturation in sub-groups such as pre-diabetes and screen-detected diabetes.

Chronic kidney hypoxia has been suggested to be a unifying pathway in the pathogenesis of diabetic kidney disease (7, 8) and renal cortical hypoxia might be particularly important as it has been shown to predict a progressive decline in renal function (9). Whether sub-clinical hypoxemia is associated with diabetic kidney disease is unknown.

This study aimed to investigate differences in blood oxygen saturation between individuals with and without diabetes with the hypothesis that persons with diabetes exhibit lower blood oxygen saturation than non-diabetic controls. Secondly, we aimed to investigate associations between blood oxygen saturation and hemoglobin A1C (HbA_1C_), albuminuria, and kidney function respectively. To provide mechanistic insight, we explored differences between groups in parameters reflecting lung function and red blood cell characteristics.

## Materials and methods

### Study design and participants

Data for the study were derived from the Lolland-Falster Health Study (LOFUS), a cohort study aiming to establish health-information on inhabitants of the Danish rural region of Lolland-Falster carried out from 8th of February 2016 to 13th of February 2020 (10). In LOFUS, persons over 18 years were randomly selected from the Danish Civil Registration System and invited along with their household. The participation rate was 36%. A detailed description of the study protocol has been published previously (10). The study was approved by Region Zealand's Ethical Committee on Health Research (SJ-421) and registered at the Danish Data Protection Agency (REG-024-2015) as well as Clinicaltrials.gov (NCT02482896). All participants provided informed written consent. The study complied with the Declaration of Helsinki and Good Clinical Practice Guidelines. Inclusion criteria for the present study were (1) Participants from LOFUS > 18 years of age; (2) who had a measurement of blood oxygen saturation. Exclusion criteria were (1) self-reported COPD; (2) self-reported asthma or asthma-medication (4) missing information on COPD or Asthma; (5) missing data on HbA_1C_.

### Study visit and measurements

Participants attended the study visit non-fasting. Physical examinations comprised measurements of blood oxygen saturation (Nellcor^TM^ PM10N, Covidien, Mansfield, USA), blood pressure (Welch Allyn Connex Pro BPO 3400, New York, USA), and lung function (MicroLab^TM^ Handheld Spirometer, Kent, United Kingdom). Blood oxygen saturation was measured once on the left index finger. Blood pressure was measured three times with a minimum of 1 min between each measurement on the left arm and an average of the two last measurements was taken. Blood oxygen saturation and blood pressure were measured with the participant in the supine body position after 5 min of rest. Room temperature was not controlled. The spirometry was performed with the participant in the standing body position with the use of a nose clip. Gender and height were entered into the software. Three sets of values were obtained for forced expiratory volume 1 second (FEV1) and forced vital capacity (FVC) and the highest values were used. The ratio of FEV1 to FVC (FEV1/FVC) was calculated. The peak expiratory flow (PEF) and the forced expiratory time (FET) were measured. At home, participants filled out an electronic questionnaire with questions on diabetes (diabetes type was not reported), asthma, chronic obstructive lung disease, medication (insulin, other diabetes medication, iron supplementation, cholesterol lowering medication), and smoking habits (11). If needed, the staff helped filling out the questionnaire at the study visit. Non-fasting blood samples were collected and analyzed at the Department of Clinical Biochemistry at Nykøbing Falster Hospital. Blood sample analyses used in this study comprised HbA_1C_, calculated low-density lipoprotein cholesterol (LDL), eGFR, iron, hemoglobin, erythrocytes, erythrocyte mean corpuscular volume (MCV), hematocrit, ferritin, and transferrin. The urine albumin-to creatinine ratio (UACR) was based on a single spot urine. The eGFR was calculated using the CKD-EPI equation. A body mass index (BMI) was calculated from height and weight. Smoking was defined as “never smoking”, “previously smoking”, or “currently smoking”.

### Outcomes and covariates

Difference in blood oxygen saturation between diabetes (known diabetes or screen-detected diabetes) and non-diabetes (including pre-diabetes) was the primary outcome. Pre-diabetes was defined as hemoglobin A_1C_ (HbA_1C_) ≥ 42 mmol/mol and <48 mmol/mol; screen-detected diabetes as no history or treatment of diabetes and HbA_1C_ ≥ 48 mmol/mol; and known diabetes as history or current treatment of diabetes. The associations between blood oxygen saturation and HbA_1C_, UACR, and eGFR respectively were the key secondary outcomes. For analyses, UACR values reported as “ <5 mg/g” were set to 1 mg/g.

### Statistical analysis

Clinical characteristics of the participants are presented as n (%), mean ± standard deviation (SD) or if skewed distributed, as medians with interquartile range. The variables with skewed distributions were log transformed (natural logarithm) before analyses and normal distribution was obtained. For clinical characteristics, we applied the Chi-squared test for categorical data and unpaired *t*-tests or analysis of covariance (ANOVA) for continuous variables. Blood oxygen saturation levels are presented as means with 95% confidence intervals. To investigate differences in blood oxygen saturation, we used unadjusted linear regression analyses. Associations were further adjusted for the presumed confounders age, gender, smoking, and BMI. To assess the association between blood oxygen saturation and the secondary outcomes, linear regression models were applied using HbA_1C_, UACR, and eGFR respectively as exposure and blood oxygen saturation as outcome. Associations were further adjusted for age, gender, smoking, BMI, and diabetes status. Standardized regression coefficients are reported. To assess if diabetes status had effect-modifying properties, we tested for interactions between indices of kidney function and diabetes status. If there was an interaction, analyses were stratified for diabetes and non-diabetes.

A power calculation was performed based on a previous study where a mean difference of 0.5% in blood oxygen saturation between persons with and without diabetes was observed (3). To demonstrate a difference of at least 0.5% and assuming a pooled standard deviation of 2.8%, at least 660 participants in each group were required to demonstrate a significant difference between groups with an alpha of 5% and a power of 90%. Assuming a diabetes prevalence of 5% in a Danish population (12), we estimated that the LOFUS cohort of 16.084 participants would encompass ~804 persons with diabetes allowing for an amply powered study.

## Results

### Clinical characteristics

**Figure 1:**
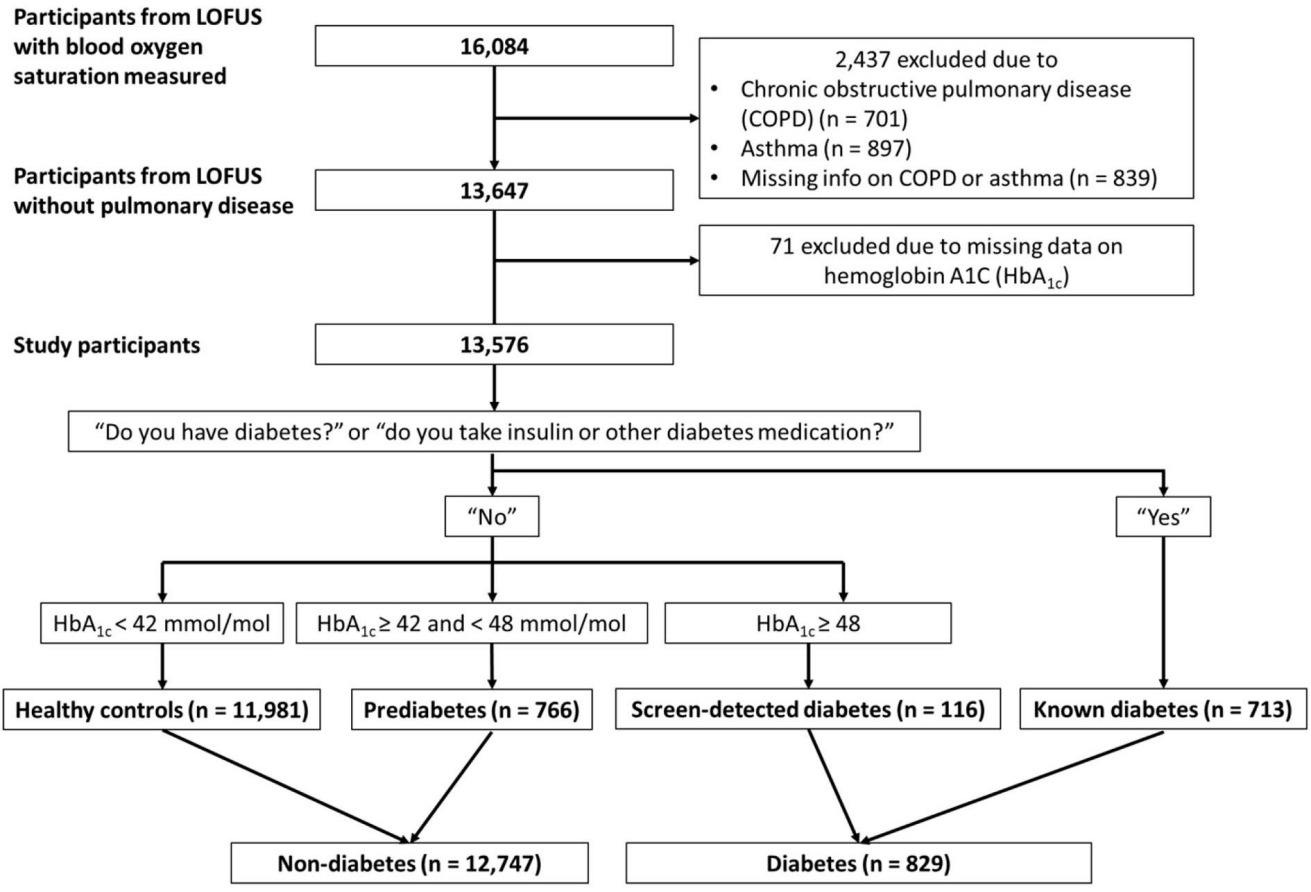
Flowchart over study participants.

There were 16,084 participants in LOFUS who had a measurement of blood oxygen saturation available. We excluded 2,437 participants with COPD (*n* = 701), asthma (*n* = 897), or missing information on these (*n* = 839). Moreover, we excluded 71 participants because of missing data on HbA_1C_, leaving a total of 13,576 participants in the present study. Of these, 12,747 had non-diabetes (94% healthy controls; 6% pre-diabetes) and 829 had diabetes (86% known diabetes; 14% screen-detected diabetes) (Figure 1).

Compared with non-diabetes, participants with diabetes were older, primarily of male gender, and had a higher BMI. Blood hemoglobin was not different when comparing the groups (Table 1).

**Table 1:** Clinical characteristics.

	**Non-diabetes**	**Diabetes**	**p**
*n*	12,747	829	
Age (years)	56 ± 16	66 ± 11	<0.001
Women	6,912 (54%)	314 (38%)	<0.001
Smoking	-	-	<0.001
Never smokers	6,120 (48%)	312 (38%)	-
Previous smokers	4,259 (34%)	366 (44%)	-
Current smokers	2,328 (18%)	149 (18%)	-
Body mass index (kg/m^2^)	27.0 ± 4.9	30.8 ± 5.5	<0.001
Systolic blood pressure (mmHg)	133 ± 19	138 ± 18	<0.001
Diastolic blood pressure (mmHg)	79 ± 8	80 ± 8	0.03
Blood oxygen saturation (%)	97.2 ± 1.7	96.4 ± 1.6	<0.001
Pre-diabetes	766 (6%)	-	-
Known diabetes	-	713 (86%)	-
Screen-detected diabetes	-	116 (14%)	-
HbA_1C_ (mmol/mol)	36 ± 4	54 ± 13	-
HbA_1C_ (%)	5.4 ± 0.3	7.1 ± 1.2	-
Blood glucose (mmol/L)	5.8 ± 1.0	8.9 ± 3.4	-
Estimated glomerular filtration rate (ml min^−1^ 1.73 m^−2^)	93 ± 16	86 ± 19	<0.001
Urine albumin creatinine ratio (mg/g creatinine)	8 [1–16]	17 [8–42]	<0.001
Hemoglobin (mmol/mol)	8.8 ± 0.7	8.8 ± 0.8	0.93
LDL cholesterol (mmol/L)	2.9 ± 0.9	2.2 ± 1	<0.001
Coronary artery disease	304 (2%)	80 (10%)	<0.001
Kidney disease	134 (1%)	35 (4%)	<0.001
Insulin treatment	-	170 (21%)	-
Other diabetes medication	-	536 (65%)	-
Iron supplement	647 (5%)	67 (8%)	<0.001
Cholesterol lowering medication	1,503 (12%)	509 (62%)	<0.001

Shown are means ± SD's, n (%) and medians [Q1; Q3]. HbA_1C_, Hemoglobin A_1C_. P-values from unpaired t-tests or chi-squared tests.

Clinical characteristics stratified in healthy controls and diabetes sub-groups (pre-diabetes, known diabetes, screen-detected diabetes) are shown in Table 2.

**Table 2:** Clinical characteristics in sub-groups.

	**Healthy controls**	**Pre-diabetes**	**Screen-detected diabetes**	**Known diabetes**	**p**
*n*	11,981	766	116	713	
Age (years)	55 ± 16	65 ± 11	64 ± 11	66 ± 11	<0.001
Women	6,507 (54%)	405 (53%)	43 (37%)	271 (38%)	<0.001
Smoking	-	-	-	-	<0.001
Never smokers	5,829 (49%)	291 (38%)	50 (43%)	262 (37%)	-
Previous smokers	3,961 (33%)	298 (39%)	45 (39%)	321 (45%)	-
Current smokers	2,158 (18%)	170 (22%)	21 (18%)	128 (18%)	-
Body mass index (kg/m^2^)	26.8 ± 4.8	29.6 ± 5.3	32.5 ± 5.8	30.6 ± 5.4	<0.001
Systolic blood pressure (mmHg)	132 ± 19	137 ± 18	142 ± 17	138 ± 18	<0.001
Diastolic blood pressure (mmHg)	79 ± 8	81 ± 8	84 ± 9	79 ± 8	<0.001
Blood oxygen saturation (%)	97.3 ± 1.7	96.3 ± 1.7	96.0 ± 1.5	96.5 ± 1.6	<0.001
HbA_1C_ (mmol/mol)	35 ± 3	43 ± 1	58 ± 16	53 ± 12	-
HbA_1C_ (%)	5.4 ± 0.3	6.1 ± 0.1	7.5 ± 1.5	7 ± 1.1	-
Blood glucose (mmol/L)	5.8 ± 0.9	6.5 ± 1.3	9.6 ± 3.8	8.7 ± 3.3	-
Estimated glomerular filtration rate (ml min^−1^ 1.73 m^−2^)	93 ± 16	80 ± 13	91 ± 15	86 ± 20	<0.001
Urine albumin creatinine ratio (mg/g creatinine)	7 [1–16]	11 [5–26]	15 [6–30]	18 [8–45]	<0.001
Hemoglobin (mmol/mol)	8.8 ± 0.7	8.8 ± 0.8	9.1 ± 0.7	8.7 ± 0.8	<0.001
LDL cholesterol (mmol/L)	2.9 ± 0.9	2.9 ± 1.1	2.9 ± 1	2.1 ± 0.9	<0.001
Coronary artery disease	249 (2%)	55 (7%)	11 (9%)	69 (10%)	<0.001
Kidney disease	118 (1%)	16 (2%)	2 (2%)	33 (5%)	<0.001
Insulin	-	-	-	170 (24%)	-
Other diabetes medication	-	-	-	536 (75%)	-
Iron supplement	617 (5%)	30 (4%)	6 (5%)	61 (9%)	<0.001
Cholesterol lowering medication	1,270 (11%)	233 (31%)	34 (31%)	475 (67%)	<0.001

Shown are means ± SD's, n (%) and medians [quartile 1–quartile 3]. HbA_1C_, Hemoglobin A1C. P-values from ANOVA and chi-squared tests.

### Primary outcome—The effect of diabetes on blood oxygen saturation

**Figure 2:**
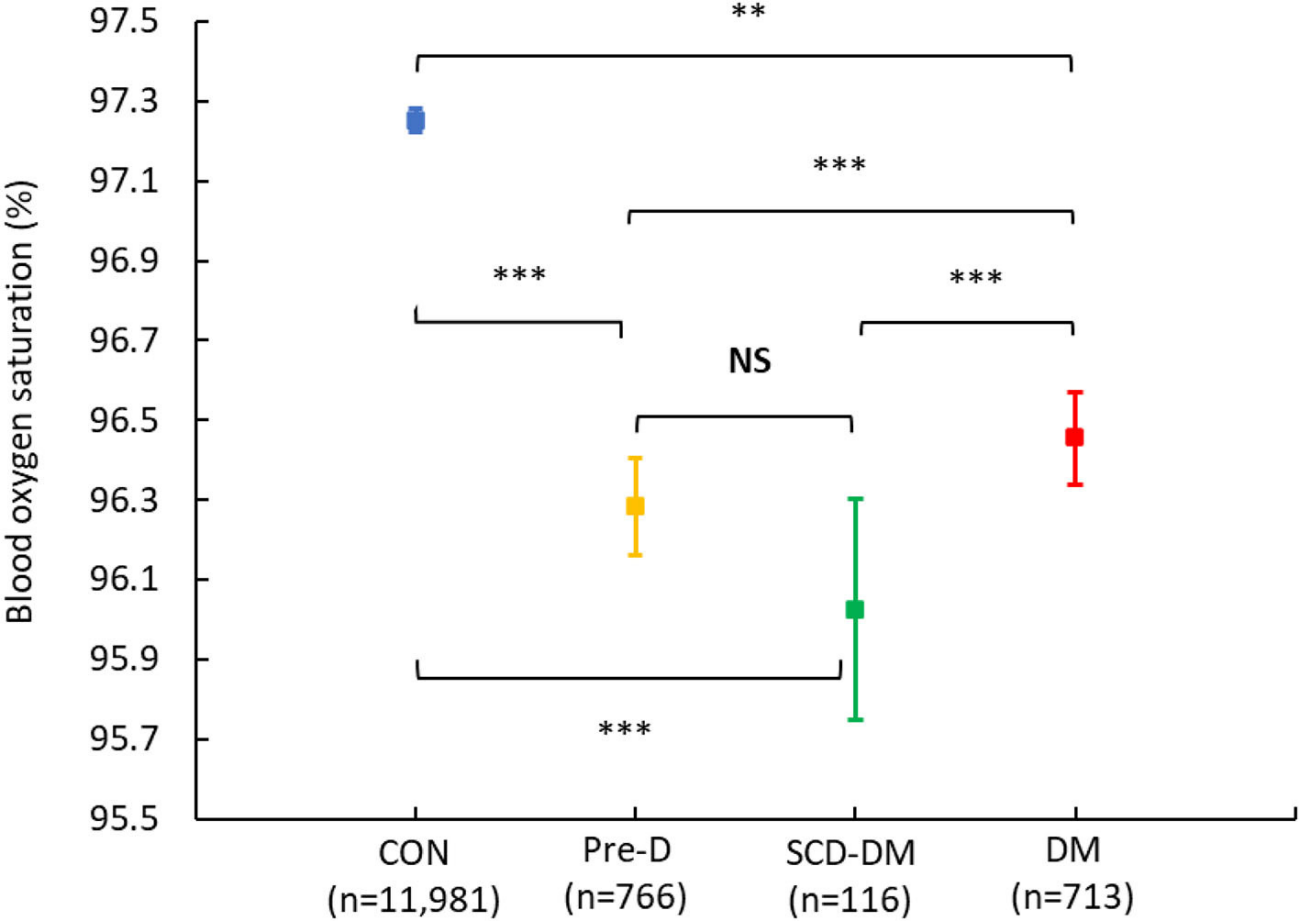
Blood oxygen saturation in sub-groups. Shown are means with 95% confidence intervals in blood oxygen saturation in participants without diabetes and pre-diabetes (CON; blue), with pre-diabetes (Pre-D; yellow), with screen-detected diabetes (SCD-DM; green), and with known diabetes (DM; red). *P*-values for between-group comparisons are calculated with the use of a linear regression analysis with diabetes-group (Non-DM; Pre-D; SCD-DM; DM) as the exposure and blood oxygen saturation as the outcome. NS, non-significant (unadjusted). **Significant after adjustment for age, gender, and smoking. ***Significant after adjustment for age, gender, smoking and body mass index (BMI).

The mean (95% CI) blood oxygen saturation was 96.3% (96.3–96.4%) for the diabetes group and 97.3% (97.2–97.3%) for the non-diabetes group, which corresponds to an unadjusted mean difference (95% CI) of −0.9% (−1.0% to −0.8%, *p* < 0.001) and −0.4% (−0.5% to −0.3%, *p* < 0.001) after adjustment for age, gender, and smoking. The difference lost significance after further adjusting for BMI (*p* = 0.26). Sub-group analyses showed that healthy controls had the highest blood oxygen saturation [97.3% (97.2–97.3%)] and that the lowest mean blood oxygen saturations were in pre-diabetes [96.3% (96.2–96.4%)] and screen-detected diabetes [96.0% (95.7–96.3%)], followed by known diabetes [96.5% (96.3–96.6%)]. Compared with healthy controls, the pre-diabetes, screen-detected diabetes, and known diabetes groups all had lower blood oxygen saturation after adjusting for age, gender, and smoking, (*p*-values < 0.001). After further adjustment for BMI, the differences between healthy controls and the pre-diabetes and screen-detected diabetes groups respectively remained significant (*p*-values < 0.01), but the difference between healthy controls and known diabetes lost significance (*p* = 0.42) (Figure 2).

### Secondary outcomes

In all participants pooled, a lower blood oxygen saturation was associated with a higher HbA_1C_ (β per 1 SD increase = −0.39% decrease (95%CI −0.41% to −0.36%); *p* < 0.001) in the unadjusted model. The association remained significant after adjusting for age, gender, and smoking (β per 1 SD increase = −0.20% (95%CI −0.23% to −0.18%); *p* < 0.001), and also after adding BMI (β per 1 SD increase = −0.11% (95%CI −0.14% to −0.08%); *p* < 0.001) (Supplementary Figure S1).

No modifying effect of diabetes status (diabetes vs. non-diabetes) on the association between UACR and blood oxygen saturation was found (*p* for interactio*n* = 0.86) and thus only pooled analyses on UACR were performed. A lower blood oxygen saturation was associated with a higher UACR (β per 1 SD increase in UACR = −0.17% (−0.20% to −0.14%; *p* < 0.001), also after adjusting for age, gender, smoking, and diabetes status (β per 1 SD increase in UACR = −0.05% (−0.08% to −0.2%; *p* < 0.001), and also after adding BMI to the model (β per 1 SD increase in UACR = −0.03% (−0.06% to −0.0%; *p* < 0.001) (Supplementary Figure S2).

A modifying effect of diabetes status on the association between eGFR and blood oxygen saturation was found (*p* for interaction < 0.001), so participants were analyzed separately in the diabetes and the non-diabetes. In the diabetes group, there was no significant association between eGFR and blood oxygen saturation (β per 1 SD increase in eGFR = 0.07% (−0.02% to 0.16%; *p* = 0.14). In the non-diabetes group, a higher blood oxygen saturation was associated with a higher eGFR (β per 1 SD increase in eGFR = 0.36% (0.33–0.39%; *p* < 0.001). After adjusting for age, gender, and smoking, the association was inversed, and a lower blood oxygen saturation was associated with a higher eGFR (β per 1 SD increase in eGFR = −0.06% (−0.9% to −0.02%; *p* < 0.001). Adding BMI did not change the association markedly (β per 1 SD increase in eGFR = −0.09% (−0.13% to −0.05%; *p* < 0.001).

### Exploratory analyses

Women had a higher blood oxygen saturation than men and smokers had a lower blood oxygen saturation than never-smokers. Higher age and BMI both associated with lower blood oxygen saturation (Supplementary Table S1). All lung function parameters except FET were impaired in the diabetes group compared with non-diabetes, including lower values in FEV1, FVC, and FEV1/FVC (Supplementary Table S2). Lung function stratified in sub-groups can be found in Supplementary Table S3. Higher blood oxygen saturation was associated with higher values in FEV1, FVC, and FEV1/FVC, and with lower values in FET (Supplementary Table S1). Plasma iron, ferritin, and transferrin were higher in the diabetes group compared with non-diabetes (Supplementary Table S4). Sub-group analyses revealed that hemoglobin, total erythrocytes, hematocrit, and ferritin were higher in the screen-detected diabetes sub-group compared with the other groups (Supplementary Table S5). Lower blood oxygen saturation was associated with higher values in hemoglobin, total erythrocytes, hematocrit, and ferritin (Supplementary Table S1).

### Sensitivity analyses

Sensitivity analyses were performed on primary and secondary outcomes by separately excluding current smokers (*n* = 2,477) and participants with kidney disease (*n* = 169). Results were confirmatory.

We did additional exploratory adjustments for FEV1 and FVC in the model of the difference between non-diabetes and diabetes in blood oxygen saturation. This did not change the estimates or significance of the differences, except for the difference between healthy controls and screen-detected diabetes in blood oxygen saturation adjusted for age, gender, smoking, BMI, FEV1, and FVC, which lost significance [−0.3% (−0.6% to 0.0%, *p* = 0.07)].

BMI and diabetes status were highly correlated: with healthy controls as a reference (26.8 ± 4.8 kg/m^2^), BMI was 2.8 kg/m^2^ (95%CI 2.5 to 3.2; *p* < 0.001) higher in pre-diabetes, 5.7 kg/m^2^ (4.8–6.6; *p* < 0.001) higher in screen-detected diabetes, and 3.7 kg/m^2^ (3.4 to 4.1; *p* < 0.001) higher in known diabetes.

## Discussion

### Principal findings

The main finding in this population-based cross-sectional study in persons with and without diabetes, was that blood oxygen saturation compared with healthy controls was lower in known diabetes, but even lower in pre-diabetes and screen-detected diabetes. For known diabetes, the difference lost significance after adjustment for BMI, but differences remained significant in pre-diabetes and screen-detected diabetes. Lower blood oxygen saturation was associated with higher HbA_1C_, a marker of dysglycemia, and with higher albuminuria, a marker of generalized microvascular dysfunction. We found no association between blood oxygen saturation and eGFR. Exploratory analyses revealed that higher age, male gender, and BMI were all associated with lower blood oxygen saturation. Furthermore, participants with diabetes had worse lung function compared with non-diabetic controls, and this was associated with lower blood oxygen saturation.

#### Primary outcome

Compared with healthy controls, blood oxygen saturation was lower in known diabetes, but even lower in screen-detected diabetes and pre-diabetes. Blood oxygen saturation was inversely associated with HbA_1C_ and since the screen-detected diabetes sub-group had the highest HbA_1C_, this association could in part explain the low blood oxygen saturation observed. The pre-diabetes sub-group however, also had lower blood oxygen saturation compared with the known diabetes sub-group and the explanation for this remain unclear. We noticed that lung function parameters (FEV1, FEV1/FVC, PEF, and FET) were most impaired in the pre-diabetes sub-group, and this could be one explanation for a lower oxygen saturation in this group. The pre-diabetes and screen-detected diabetes sub-groups had the lowest blood oxygen saturations, and these groups have in common that they have dysglycemia but receive no diabetes treatment. This could indicate that diabetes treatment has a beneficial effect on blood oxygen saturation, but longitudinal studies are needed to examine this hypothesis.

Two major causes for the lower oxygen saturation observed in the present study could be: a lower oxygen supply or a higher oxygen consumption.

##### Impaired oxygen supply

Lung function was sub-clinically impaired in participants with diabetes, confirming previous findings in type 2 diabetes (6, 13), and in a diabetes meta-analysis (14). Impaired lung function might mechanically reduce airway oxygen availability, although we did find one study in cerebral palsy showing no association between lung function and blood oxygen saturation (15). BMI was associated with lower blood oxygen saturation and might also reduce airway oxygen availability by altering lung mechanics (16). Of notice, the difference in blood oxygen saturation between non-diabetes and known diabetes lost significance after adjusting for BMI, but this is likely due to co-linearity between BMI and diabetes status, which was demonstrated in this population. In this study, we did not measure the pulmonary oxygen diffusion capacity, but previous studies indicate that this is reduced both in type 1 and type 2 diabetes, probably due to pulmonary microvascular damage (17, 18). Our finding that lower blood oxygen saturation was associated with albuminuria, which we consider a proxy for generalized microvascular damage, supports this theory. Studies investigating the association between hypoxia and microvascular complications in diabetes are warranted.

We observed no differences in red blood cell parameters indicative of an impaired red blood cell function in diabetes or pre-diabetes. On the contrary, we observed that the sub-group with screen-detected diabetes, who also had the lowest blood oxygen saturation of all groups, had higher values in plasma hemoglobin, total erythrocytes, hematocrit, and ferritin compared with the other groups. The most likely explanation is an adaptive increase in response to sub-clinical hypoxemia (19).

##### Oxygen consumption

A higher BMI is associated with a higher myocardial oxygen consumption (20) and since the heart is a highly metabolic organ (21), this might influence the systemic balance between oxygen supply and consumption, reducing the overall blood oxygen saturation in those with higher BMI, i.e. the pre-diabetes -, screen-detected diabetes-, and known diabetes sub-groups. At the same time, the kidneys consume large amounts of oxygen, primarily for reabsorbing glucose and sodium from the glomerular filtrate in the proximal tubules. Relative hyperglycemia in the diabetes group might thus cause an increased kidney oxygen consumption *via* increased proximal tubule glucose reabsorption.

#### Previous work on blood oxygenation in diabetes

Three studies in type 1 diabetes have demonstrated an ~0.5% lower blood oxygen saturation compared with non-diabetic controls (2, 3, 22). One study with 26 persons with type 2 diabetes and 24 non-diabetic controls showed a 0.3% lower blood oxygen saturation in the diabetes group, but the difference was not significant (4). Another study monitored blood oxygen saturation for a minimum of 5 h in obese women with (*n* = 30) and without (*n* = 60) type 2 diabetes and demonstrated no significant difference between groups in the mean blood oxygen saturation, but interestingly found that the amount of time spent with low blood oxygen saturation (<90%) was 20% in the diabetes group, which was three-times higher than the 7% reported for the non-diabetic control group (23). A large study in 470 persons with type 2 diabetes and 4,402 non-diabetic controls similarly found that the time spent with blood oxygen saturation <90% during sleep was higher in diabetes compared with non-diabetes, but mean blood oxygen saturation levels were not reported (24). A very important difference between the previous studies mentioned and the present study is that information on diabetes type was not available in our study. We however presume that the diabetes group in this study consisted ~90% of type 2 diabetes (12). Another difference is that blood oxygen saturation was not measured the same way in previous studies and in the present study, regarding the number of measurements and recording time. The present study sample size is however larger than in previous studies and the differences observed are independent of age, gender, and smoking.

#### Implications and further studies

This work supports the hypothesis that blood oxygen saturation is lower in persons with diabetes compared with non-diabetic controls and reveals that persons with pre-diabetes and screen-detected diabetes have sub-clinical hypoxemia. This could be clinically important for several reasons:

1. Sub-clinical hypoxemia might be a consequence of a combination of problems, including pulmonary microvascular dysfunction and respiratory autonomic neuropathy (3). Blood oxygen saturation could thus potentially be utilized as a biomarker for early screening for these conditions or to monitor drug effects in clinical trials. Pulse oximetry may not be the best method, but new blood oxygen saturation remote monitoring devices have emerged recently and these could be feasible for such purposes (25). Importantly, these methods allow for blood oxygen saturation monitoring for longer periods of time, for example for 24 h.
2. Our findings are a small step in improving the general understanding of the role of blood oxygen saturation in the pathophysiology of diabetic complications, but studies are needed to determine whether sub-clinical hypoxemia plays an independent role in the progression of diabetic complications such as kidney disease, retinopathy, and neuropathy, or if sub-clinical hypoxemia is merely a consequence or symptom of diabetic complications.
3. Low blood oxygen saturation is associated with higher mortality (1) and our findings could be preliminary for mechanistic studies or clinical trials investigating interventions to improve blood oxygen saturation in persons with diabetes. In this context, we and others have shown that increasing blood oxygen saturation acutely by breathing exercises improve cardiovascular autonomic function in persons with diabetes (4, 26, 27). The development of feasible interventions to improve blood oxygen saturation chronically and studies investigating their effects on cardiovascular autonomic function in diabetes are thus warranted.

#### Strengths and weaknesses

There is a risk of selection bias with the participation rate in LOFUS of 36% and furthermore when excluding participants without a valid pulse oximetry measurement or with COPD or asthma. The diabetes type is unknown, which is a limitation that reduces the external validity of our findings, but we assume that most participants had type 2 diabetes (12). Pulse oximetry was only performed once which is a limitation (low precision) when comparing with previous studies that performed pulse oximetry measurements once each second over a period of 5 min and taking an average (2, 3, 22), or for even longer (23). BMI was highly correlated with diabetes status and this co-linearity could explain the loss of significance when adding BMI to the models. It is a weakness that we lacked data on conditions besides COPD and asthma, which might have impacted the study outcomes, such as interstitial lung disease and non-diabetic kidney disease. Room temperature was not controlled during measurements of blood oxygen saturation, which might have given erroneously lower readings on cold days.

The primary strength of this study is the large sample size, which gave power for stratified analyses and sub-group analyses.

## Conclusion

In this population-based cross-sectional study with 829 persons with diabetes and 12.747 non-diabetic controls, we found that blood oxygen saturation was lower in persons with known diabetes compared with healthy controls and even lower in sub-groups with pre-diabetes and screen-detected diabetes. These findings were independent of age, gender, and smoking. For known diabetes, the difference lost significance after adjustment for BMI, but differences remained significant in pre-diabetes and screen-detected diabetes. The loss of significance after adding BMI is likely due to the observed co-linearity between BMI and diabetes status. Lower blood oxygen saturation was associated with dysglycemia and with higher albuminuria, a marker of generalized microvascular dysfunction, but we found no association with kidney function. Although both lung function and BMI seemed important for blood oxygen saturation, the exact mechanisms are still unknown and longitudinal studies to investigate causal relations are warranted.

## Data availability statement

Individual, de-identified participant data are not freely available because of the risk of patient re-identification but interested parties can request access to de-identified participant data or anonymised clinical study reports through submission of a request for access to the corresponding author, provided that the necessary data protection agency and ethical committee approvals are provided in compliance with relevant legislation.

## Ethics statement

The study was approved by Region Zealand's Ethical Committee on Health Research (SJ-421) and registered at the Danish Data Protection Agency (REG-024-2015) as well as Clinicaltrials.gov (NCT02482896). The patients/participants provided their written informed consent to participate in this study.

## Author contributions

JL and CH conceived and designed the research and performed the statistical analysis. JL drafted the manuscript. RJ and NB-R acquired the data. RJ, NB-R, MF-M, MJ, and PR contributed to the interpretation of the results and reviewed/edited the manuscript. JL is the guarantor of this work and, as such, had full access to all the data in the study and takes responsibility for the integrity of the data and the accuracy of the data analysis. All authors contributed to the article and approved the submitted version.

## Funding

The study was funded by the Novo Nordisk Foundation Grant PROTON personalizing treatment of diabetic nephropathy (NNF14OC0013659). The Lolland-Falster Health Study (LOFUS) was funded by Region Zealand, Nykøbing F. Hospital, Lolland Municipality, and Guldborgsund Municipality.

## Conflict of interest

Author JL reports having received a speaking fee from Boehringer Ingelheim, which was given to Steno Diabetes Center Copenhagen. Author PR reports having received research grants from Astra Zeneca and Novo Nordisk and given lectures for Astra Zeneca, and Boehringer Ingelheim and has served as a consultant for Astra Zeneca, Bayer, Eli Lilly, Boehringer Ingelheim, Astellas, Gilead, Sanofi Aventis Vifor, and Novo Nordisk, all fees given to Steno Diabetes Center Copenhagen. Author MF-M reports having received research grants from Novo Nordisk and speaking fees from Boehringer Ingelheim, Novartis, Baxter and Sanofi. Author MJ has received research grants from Astra Zeneca, AMGEN, Boehringer Ingelheim, Novo Nordisk, and Sanofi Aventis. The remaining authors declare that the research was conducted in the absence of any commercial or financial relationships that could be construed as a potential conflict of interest.

## Publisher's note

All claims expressed in this article are solely those of the authors and do not necessarily represent those of their affiliated organizations, or those of the publisher, the editors and the reviewers. Any product that may be evaluated in this article, or claim that may be made by its manufacturer, is not guaranteed or endorsed by the publisher.
