# Supplementary material for: Blood oxygen saturation is lower in persons with pre-diabetes and screen-detected diabetes compared with non-diabetic individuals: A population-based study of the Lolland-Falster Health Study cohort
Source: Front Epidemiol. 2022 Oct 13;2:1022342. doi: 10.3389/fepid.2022.1022342 (PMC10910962; doi:10.3389/fepid.2022.1022342)
Supplement: Supplementary file 1 [file Data_Sheet_1.docx]

**Supplementary Material**

**Contents**

[Table S1. Exploratory analyses 2](#_Toc99113383)

[Table S2. Lung function in participants with and without diabetes 3](#_Toc99113384)

[Table S3. Lung function in diabetes sub-groups 4](#_Toc99113385)

[Table S4. Hematology in participants with and without diabetes 5](#_Toc99113386)

[Table S5. Hematology in diabetes sub-groups 6](#_Toc99113387)

[Figure S1. The association between HbA_1C_ and blood oxygen saturation in all participants 7](#_Toc99113388)

[Figure S2. The association between albuminuria and blood oxygen saturation in all participants 8](#_Toc99113389)

# Table S1. Exploratory analyses

| **Parameter** | **Difference vs. reference in blood oxygen saturation (%)** | **95%CI** | ***p*** |
| --- | --- | --- | --- |
| Women vs. men | 0.46 | 0.40 to 0.51 | <0.001 |
| Current smoking vs. non-smoking | 0.07 | -0.01 to 0.14 | 0.07 |
| Current smoking vs. never-smoking | -0.32 | -0.40 to -0.24 | <0.001 |
| **Parameter** | **Change in blood oxygen saturation (%) per 1 SD increase in parameter** | **95%CI** | ***p*** |
| Age | -0.60 | -0.63 to -0.57 | <0.001 |
| BMI | -0.49 | -0.52 to -0.47 | <0.001 |
| **Lung function** |  |  |  |
| FEV1 | 0.25 | 0.21 to 0.28 | <0.001 |
| FVC | 0.19 | 0.16 to 0.22 | <0.001 |
| FEV1/FVC | 0.18 | 0.15 to 0.21 | <0.001 |
| PEF | 0.05 | 0.02 to 0.09 | <0.001 |
| FET | -0.21 | -0.25 to -0.18 | <0.001 |
| **Hematology** |  |  |  |
| Iron | -0.02 | -0.05 to 0.01 | 0.17 |
| Hemoglobin | -0.28 | -0.31 to -0.26 | <0.001 |
| Erythrocyte MCV | -0.12 | -0.15 to -0.09 | <0.001 |
| Erythrocytes | -0.18 | -0.21 to -0.15 | <0.001 |
| Hematocrit | -0.28 | -0.31 to -0.25 | <0.001 |
| Ferritin | -0.27 | -0.30 to -0.24 | <0.001 |
| Transferrin | 0.18 | 0.15 to 0.21 | <0.001 |

**Table S1. Exploratory analyses.** Shown are standardized estimates with 95% confidence intervals. BMI = body mass index, FEV1 = forced expiratory volume, FVC = forced vital capacity, PEF = peak expiratory flow, FET = forced expiratory time, MCV = mean corpuscular volume.

# Table S2. Lung function in participants with and without diabetes

|  | Non-diabetes | Diabetes | *p* |
| --- | --- | --- | --- |
|  | 12,747 | 829 | - |
| FEV1 (L) | 3.0 ± 0.9 | 2.6 ± 0.7 | <0.001 |
| FVC (L) | 3.9 ± 1.1 | 3.4 ± 0.9 | <0.001 |
| FEV1 predicted (%) | 77.1 ± 7.5 | 76.3 ± 7.5 | 0.02 |
| FVC predicted (%) | 99.9 ± 17.8 | 92.8 ± 17.4 | <0.001 |
| FEV1/FVC (%) | 106.7 ± 16.1 | 97.6 ± 17.4 | <0.001 |
| PEF (L/s). | 7.2 ± 2 | 6.8 ± 1.9 | <0.001 |
| PEF predicted (%) | 97.0 ± 18.7 | 92.7 ± 18.9 | <0.001 |
| Forced Expiratory Time (s) | 6.3 ± 2.4 | 6.4 ± 2.4 | 0.28 |

**Table S2. Lung function in participants with and without diabetes.** Shown are means ± SD’s. *P-*values from unpaired ttests. FEV1 = forced expiratory volume, FVC = forced vital capacity, PEF = peak expiratory flow, FET = forced expiratory time.

# Table S3. Lung function in diabetes sub-groups

|  | Non-diabetes | Prediabetes | Screen-detected Diabetes | Known diabetes | *p* |
| --- | --- | --- | --- | --- | --- |
|  | 11,981 | 766 | 116 | 713 | - |
| FEV1 (L) | 3.0 ± 0.9 | 2.5 ± 0.7 | 2.6 ± 0.7 | 2.6 ± 0.7 | <0.001 |
| FVC (L) | 3.9 ± 1.1 | 3.4 ± 0.9 | 3.4 ± 1 | 3.4 ± 0.9 | <0.001 |
| FEV1 predicted (%) | 100 ± 18 | 93 ± 17 | 96 ± 18 | 92 ± 18 | <0.001 |
| FVC predicted (%) | 107 ± 16 | 98 ± 17 | 103 ± 18 | 97 ± 17 | <0.001 |
| FEV1/FVC (%) | 77.1 ± 7.4 | 75.5 ± 7.8 | 76.7 ± 7.6 | 76.3 ± 7.5 | <0.001 |
| PEF (L/s). | 7.2 ± 2 | 6.5 ± 1.9 | 6.9 ± 1.9 | 6.8 ± 1.9 | <0.001 |
| PEF predicted (%) | 97 ± 19 | 93 ± 20 | 94 ± 19 | 92 ± 19 | <0.001 |
| Forced Expiratory Time (s) | 6.2 ± 2.4 | 6.5 ± 2.4 | 6.4 ± 2.7 | 6.4 ± 2.3 | 0.02 |

**Table S3. Lung function in** **diabetes sub-groups.** Shown are means ± SD’s. *P-*values from analysis of covariance (ANOVA). FEV1 = forced expiratory volume, FVC = forced vital capacity, PEF = peak expiratory flow, FET = forced expiratory time.

# Table S4. Hematology in participants with and without diabetes

|  | Reference | Non-diabetes | Diabetes | *p* |
| --- | --- | --- | --- | --- |
| N | - | 12,747 | 829 | - |
| Iron (µmol/l) | 9 - 34 | 16 ± 6 | 15 ± 5 | <0.001 |
| Hemoglobin (mmol/L) | 7.3 - 9.5 | 8.8 ± 0.7 | 8.8 ± 0.8 | 0.93 |
| Erythrocyte MCV (fL) | 82 - 98 | 91 ± 5 | 91 ± 5 | 0.20 |
| Erythrocytes (×10^12^/L) | 3.9 - 5.2 | 4.7 ± 0.4 | 4.7 ± 0.4 | 0.40 |
| Hematocrit (%) | 35 – 46 | 43 ± 3 | 43 ± 4 | 0.10 |
| Ferritin (µg/L) | 42 - 300 | 129 ± 125 | 151 ± 159 | <0.001 |
| Transferrin (g/L) | 1.91 – 3.26 | 2.64 ± 0.40 | 2.70 ± 0.38 | <0.001 |

**Table S4. Hematology in participants with and without diabetes.** Shown are means ± SD’s and medians [quartile 1; quartile 3]. *P-*values from unpaired ttests. MCV = mean corpuscular volume.

# Table S5. Hematology in diabetes sub-groups

|  | Reference | Non-diabetes | Prediabetes | Screen-detected Diabetes | Known diabetes | *p* |
| --- | --- | --- | --- | --- | --- | --- |
| N | - | 11,981 | 766 | 116 | 713 | - |
| Iron (µmol/l) | 9 - 34 | 16 ± 6 | 15 ± 5 | 16 ± 6 | 15 ± 5.2 | <0.001 |
| Hemoglobin (mmol/L) | 7.3 - 9.5 | 8.8 ± 0.7 | 8.8 ± 0.8 | 9.1 ± 0.7 | 8.7 ± 0.8 | <0.001 |
| Erythrocyte MCV (fL) | 82 - 98 | 91 ± 5 | 91 ± 5 | 91 ± 5 | 91 ± 5 | 0.17 |
| Erythrocytes (×10^12^/L) | 3.9 - 5.2 | 4.7 ± 0.4 | 4.8 ± 0.4 | 4.9 ± 0.4 | 4.7 ± 0.4 | <0.001 |
| Hematocrit (%) | 35 - 46 | 43 ± 3 | 43 ± 3 | 46 ± 3 | 43 ± 4 | <0.001 |
| Ferritin (µg/L) | 42 - 300 | 98 [49, 170] | 116 [62, 190] | 208 [110, 328] | 97 [52, 177] | <0.001 |
| Transferrin (g/L) | 1.91 – 3.26 | 2.64 ± 0.4 | 2.66 ± 0.38 | 2.70 ± 0.36 | 2.70 ± 0.38 | <0.001 |

**Table S5. Hematology in** **diabetes sub-groups.** Shown are means ± SD’s and medians [quartile 1; quartile 3]. *P-*values from analysis of covariance (ANOVA). MCV = mean corpuscular volume.

# Figure S1. The association between HbA_1C_ and blood oxygen saturation in all participants

**
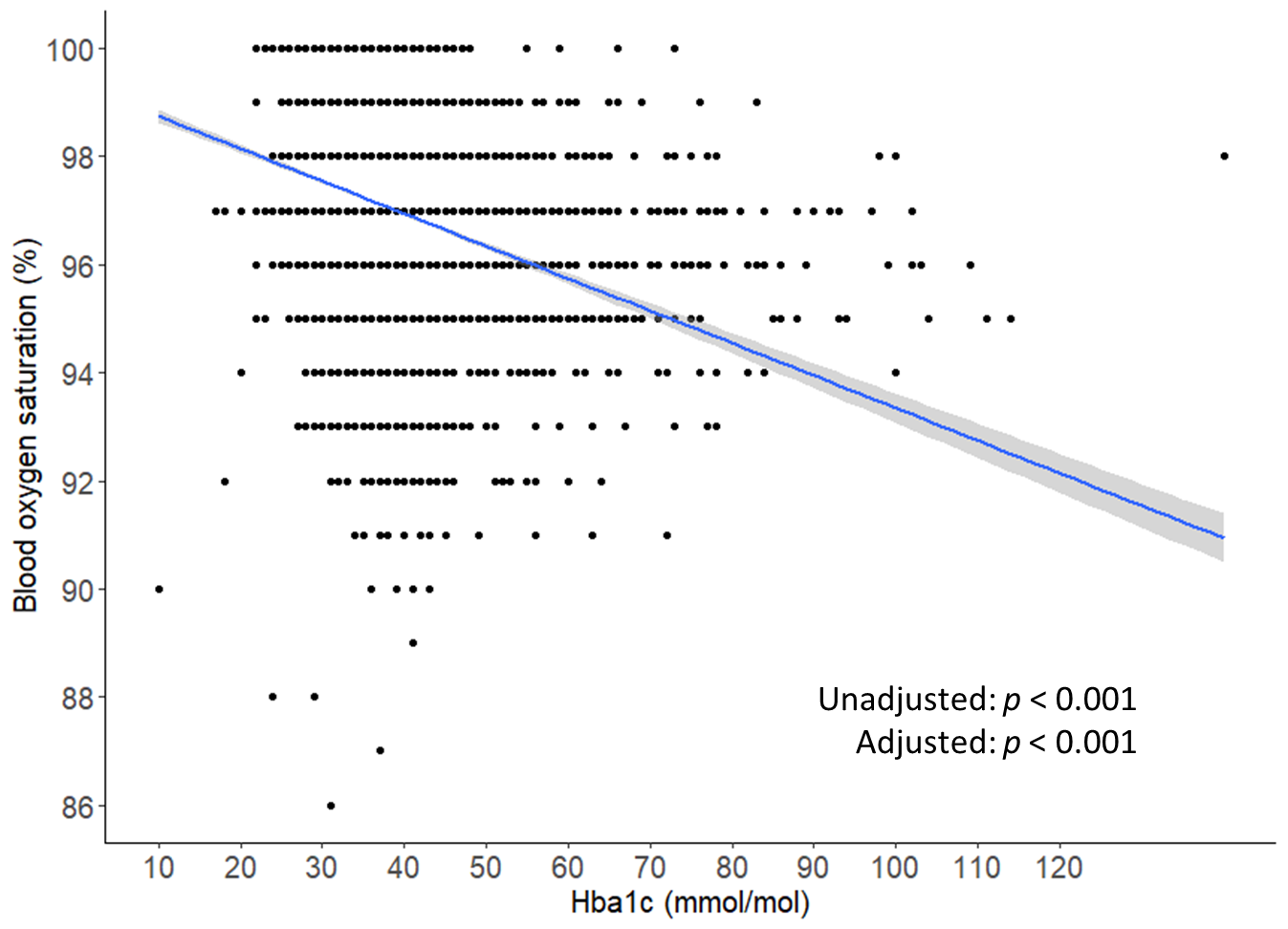
**

**Figure S1. The association between HbA_1C_ and blood oxygen saturation in all participants.** Shown is an unadjusted linear regression model with blood oxygen saturation as the outcome and Hemoglobin A_1C_ (HbA_1C_) as the exposure. *P*-values are reported from the unadjusted model and after adjusting for age, gender, current smoking, and BMI.

# Figure S2. The association between albuminuria and blood oxygen saturation in all participants

**
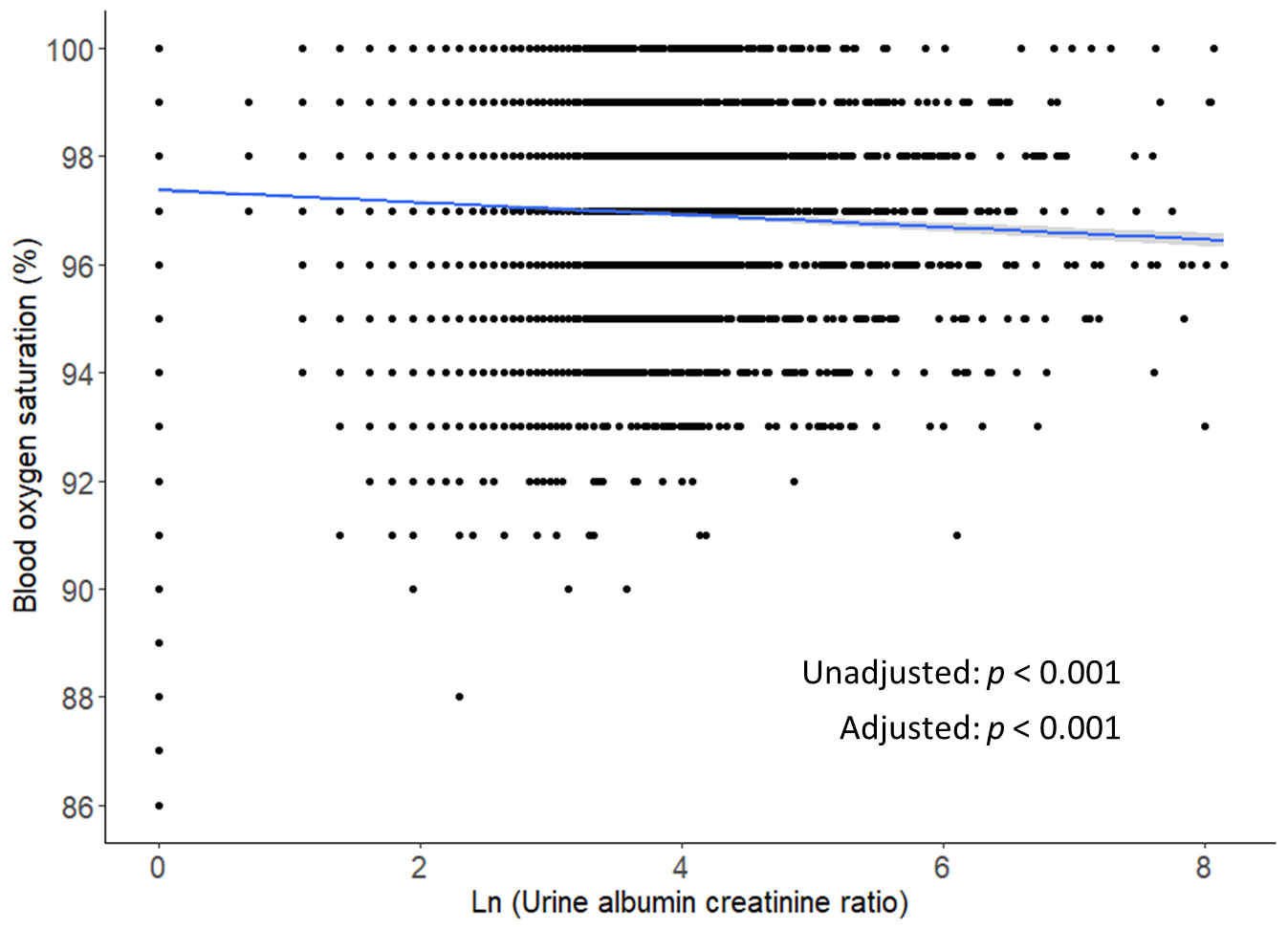
**

**Figure S2. The association between albuminuria and blood oxygen saturation in all participants.** Shown is an unadjusted linear regression model with blood oxygen saturation as the outcome and ln (urinary albumin creatinine ratio) as the exposure in all participants with a urine albumin creatinine ratio (UACR) measurement (n = 13,082). UACR was skewed and log-transformed (natural logarithm) before analyses. *P*-values are shown from the unadjusted models and after adjusting for age, gender, current smoking, and BMI.
